# De novo transcriptome analysis of *Chlorella sorokiniana*: effect of glucose assimilation, and moderate light intensity

**DOI:** 10.1038/s41598-020-74410-4

**Published:** 2020-10-15

**Authors:** Siti Nor Ani Azaman, Darren C. J. Wong, Sheau Wei Tan, Fatimah M. Yusoff, Norio Nagao, Swee Keong Yeap

**Affiliations:** 1 Centre of Foundation Studies for Agricultural Sciences, Universiti Putra Malaysia, Serdang, Selangor Malaysia; 2 Aquatic Animal Health and Therapeutics Laboratory (AquaHealth), Institute of Bioscience, Universiti Putra Malaysia, Serdang, Selangor Malaysia; 3 Ecology and Evolution, Research School of Biology, The Australian National University, Canberra, ACT 2600 Australia; 4 Laboratory of Vaccine and Biomolecules (VacBio), Institute of Bioscience, Universiti Putra Malaysia, Serdang, Selangor Malaysia; 5 International Institute of Aquaculture and Aquatic Sciences (I-AQUAS), Universiti Putra Malaysia, Port Dickson, Negeri Sembilan Malaysia; 6 Department of Aquaculture, Faculty of Agriculture, Universiti Putra Malaysia, Serdang, Selangor Malaysia; 7 102 Naname-go, Shinkamigoto-cho, Minami Matsuura-Gun, Nagasaki 857-4214 Japan; 8 China-ASEAN College of Marine Sciences, Xiamen University Malaysia, Sepang, Selangor Malaysia

**Keywords:** Transcriptomics, Gene expression, RNA sequencing

## Abstract

*Chlorella* can produce an unusually wide range of metabolites under various nutrient availability, carbon source, and light availability. Glucose, an essential molecule for the growth of microorganisms, also contributes significantly to the metabolism of various metabolic compounds produced by *Chlorella*. In addition, manipulation of light intensity also induces the formation of secondary metabolites such as pigments, and carotenoids in *Chlorella*. This study will focus on the effect of glucose addition, and moderate light on the regulation of carotenoid, lipid, starch, and other key metabolic pathways in *Chlorella sorokiniana*. To gain knowledge about this, we performed transcriptome profiling on *C. sorokiniana* strain NIES-2168 in response to moderate light stress supplemented with glucose under mixotrophic conditions. A total of 60,982,352 raw paired-end (PE) reads 100 bp in length was obtained from both normal, and mixotrophic samples of *C. sorokiniana*. After pre-processing, 93.63% high-quality PE reads were obtained, and 18,310 predicted full-length transcripts were assembled. Differential gene expression showed that a total of 937, and 1124 genes were upregulated, and downregulated in mixotrophic samples, respectively. Transcriptome analysis revealed that the mixotrophic condition caused upregulation of genes involved in carotenoids production (specifically lutein biosynthesis), fatty acid biosynthesis, TAG accumulation, and the majority of the carbon fixation pathways. Conversely, starch biosynthesis, sucrose biosynthesis, and isoprenoid biosynthesis were downregulated. Novel insights into the pathways that link the enhanced production of valuable metabolites (such as carotenoids in *C. sorokiniana*) grown under mixotrophic conditions is presented.

## Introduction

*Chlorella* species is one the most popular microalgae applied in many industries (especially pharmaceuticals, and health) across many Asian countries such as China, Korea, and Japan for hundreds of years^1^. The ability of this species to produce metabolites that possess various health-promoting activities such as antioxidant, antimicrobial, antibacterial, antifungal, anticancer, and antiviral activities make them popular in such industries^2–4^. The potential of producing different pigments by *Chlorella* species also paves a new way for commercial application of this species, making this system an excellent alternative to current pigment-producing microalgae species. For example, several studies have shown that pigment production was higher in *Chlorella* compared to other microalgae species^5–7^, and can serve well in commercial applications due to their adaptability to various culture conditions^8–10^.

In an attempt to induce the formation of higher metabolite production such as pigments, and lipid production, manipulation of carbon source, and light intensity were commonly used^5–7^. Carbon sources like glucose, glycerol, or acetate have contributed to increased biomass, and lipid productivity of microalgae^11,12^. Light intensity also influences microalgal growth, and the biochemical composition of cells through the process of photo-acclimation, and photo-adaptation^13,14^. In photo-adaptation, the microalgae change their fatty acid compositions, pigment composition, growth rate, and dark respiration rate^13^. For example, an increase in light intensity led to an increase in chlorophyll a, and other light-harvesting pigments (such as primary carotenoids, chlorophyll b, chlorophyll c, and phycobiliproteins)^14^. On the other hand, when microalgae were exposed to higher light intensity, chlorophyll a, and other pigments directly involved in photosynthesis were reduced, while carotenoids, which serve as photo-protective agents, such as zeaxanthin, β-carotene, and astaxanthin were found to be increased. However, saturated light intensity, may also disrupt the chloroplast lamellae, and inactivate enzymes involved in carbon dioxide fixation, resulting in photo-inhibition^15^.

Although a lot of studies were reported on the effect of various cultivation condition on metabolite production in microalgae^16–18^, a more specific analysis on the effect of genes related to physiological changes were still unpromising. Most were species-specific, and limited to certain treatments only^19–21^. On the other hand, genomic study provides very wide coverage of genetic information on the biological systems of microalgae. For example, Hovde et al*.*^22^ who studied the genomic characteristics of similar *Chlorella* species of three different strains showed a significant divergence on their gene content, and nucleotide identity. They also found high variability in transcriptional regulation between the three strains due to distinctive epigenetic machinery. These findings also suggest that each strain had adapted their genomic content according to the new maintained environment. Therefore, individual assessment of different strain is potentially required for understanding their performance in cultivation systems to improve the biomass, and bioproduct yields. In the other genomic report, there are highly conserved genes among the C*hlorella* species found in the chloroplast providing information that could be applied in microalgal systematics, phylogenetic reconstruction, and biotechnology^23^. In the construction of a microalgal system for commercial application, transcriptomic study could be more efficient, and relatively economical compared to genomic study^24^. It provides an initial, and broad view of molecular, and biochemical mechanisms related to the treatment, and condition used^21,25,26^. Moreover, a growing number of transcriptomes of microalgae were sequenced, assembled, and annotated, providing information on how genes are regulated, and reveal details of the microalgal biology^27–30^.

In our previous study, we examined the biochemical changes, and the morphology of microalgae *Chlorella* s*orokiniana* under photoautotrophic, and mixotrophic conditions^31^. We found that glucose, and moderate light intensity treatments contributed to a higher scavenging activity, key changes in pigmentation (i.e.shifts from chlorophyll to carotenoids), and accumulation of lipid bodies compared to the normal light condition. To date, molecular insights into the combinatorial effect of glucose, and light intensity in microalgae, or specifically in *Chlorella* species is lacking. Thus, this study explored the effect of mixotrophic condition (i.e. moderate light intensity supplemented with 2% glucose under nutrient-limited condition) on *C. sorokiniana* using transcriptomics. Several novel findings on the genes involved, and their related pathways, especially the production of secondary metabolites, are highlighted.

## Results

### Illumina sequencing, and de novo assembly

The transcriptome of *C. sorokiniana* grown under normal, and mixotrophic conditions generated from RNA with RIN > 7.8 was sequenced using Illumina sequencing platform. A total of 60,982,352 raw paired-end (PE) reads 100 bp in length were obtained from both normal, and mixotrophic samples of *C. sorokiniana*. After read trimming, and quality filtering, 57,097,573 (93.63%) high-quality PE reads were obtained (Table 1). A total of 102,643 contigs were successfully assembled using Trinity software^32^. *Bonafide* transcripts (e.g. complete genes containing both start, and stop codons) were identified using AUGUSTUS resulting in a final assembly of 18,310 full-length contigs (defined as transcripts from here on in). The transcripts ranged from 165 to 16,695 bp in length with the mean length of 1191 bp, and an N50 value of 1446 bp were obtained (Table 2). Sequencing data were deposited in Gene Expression Omnibus (GEO) at accession number GSE105427.

**Table 1:** Sequencing throughput, and trimming results for *C. sorokiniana* RNA-seq data.

Sample	Total sequences		Average length	
	Before	After	Before	After
Normal replicate 1	13,225,251	12,714,292	100	79
Normal replicate 2	16,604,976	15,073,499	100	74
Mixotrophic replicate 1	16,690,891	15,817,686	100	75
Mixotrophic replicate 2	14,461,234	13,492,097	100	75
Total	60,982,352	57,097,573	100	75

Two samples: Normal, and Mixotrophic (moderate light stress supplemented with glucose), with three biological replicates of 100 bp reads were generated on the Illumina HiSeq 2000 platform.

**Table 2:** Summary of *de-novo* assembly result for *C. sorokiniana* RNA-seq data.

Subject	Number of reads	Length (bp)
Total number of high-quality paired-end reads	57,097,573	
Number of contigs	102,643	
Number of protein-coding transcripts	18,310	
N50 (bp)		1,446
N90 (bp)		654
Longest transcript (bp)		16,695

### Functional annotation

In order to assign functional information, the transcripts were annotated through multiple databases (see “Materials and methods” section). Functional annotation using the NR database showed the highest match, in which 99.8% (or 18,276 genes) of *C. sorokiniana* transcripts had succesfull hits (Table 3). When the assembled transcripts were annotated against InterPro, Swiss Prot, Mercator Mapman, and Refseq, 50% to 55% of *C. sorokiniana* transcripts had matches, whereas, annotation to KEGG, and GO databases produced 38.1%, and 43.1% matches, respectively.

**Table 3:** Summary of the functional annotation of the assembled *C. sorokiniana* transcriptome using different databases.

Public database	No. of annotated genes	% of annotated genes
NR	18,276	99.8
InterPro	10,019	54.7
UniProt/Swiss Prot	10,259	56.0
KO	6971	38.1
GO	7896	43.1
Mapman	9341	51.0
Refseq	10,187	55.6

**Figure 1:**
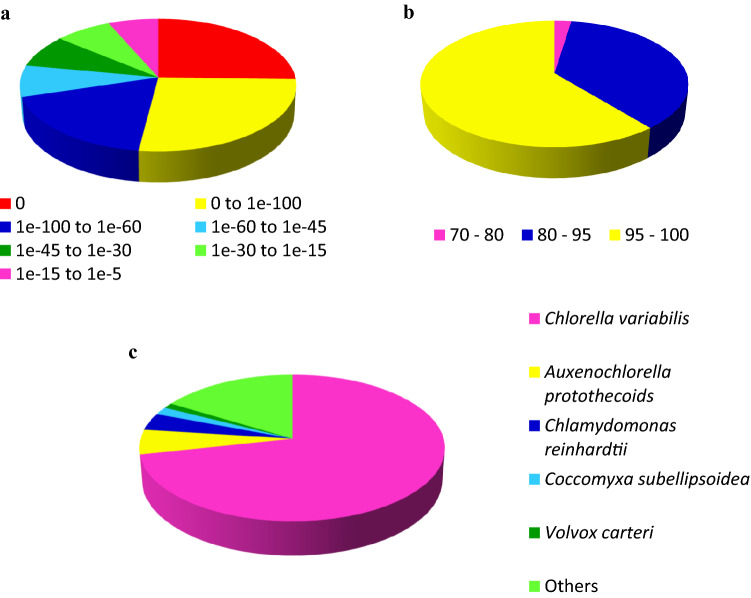
Transcript homology searches against the nr database. (**a**) The proportional frequency of the E-value distribution. (**b**) The proportional frequency of the sequence similarity distribution. (**c**) The proportional species distribution of *C. sorokiniana* transcriptome among other viridiplantae.

Based on the NR annotation result, the E-value frequency distribution analysis revealed that 70.3% of the matched sequences had strong homology with E-value ≤ 1.0E−60, while the remaining 29.7% fell into the range of 1.0E−60 to 1.0E−5 (Fig. 1). Furthermore, we also observed that all the annotated sequences had a similarity of more than 70%. Based on the homologous species identified among the annotated transcripts, 71.6% of them matched to *Chlorella variabilis*, followed by *Auxenochlorella protothecoids* (5.5%), *Chlamydomonas reinhardtii* (3.8%), *Coccomyxa subellipsoidea* (1.7%), and *Volvox carteri* (1.2%). About 16.3% of the transcripts had similarity matches with other types of viridiplantae. In addition, Mercator determined that 9341 transcripts (51%) had at least one ascribed Mapman annotation.

**Figure 2:**
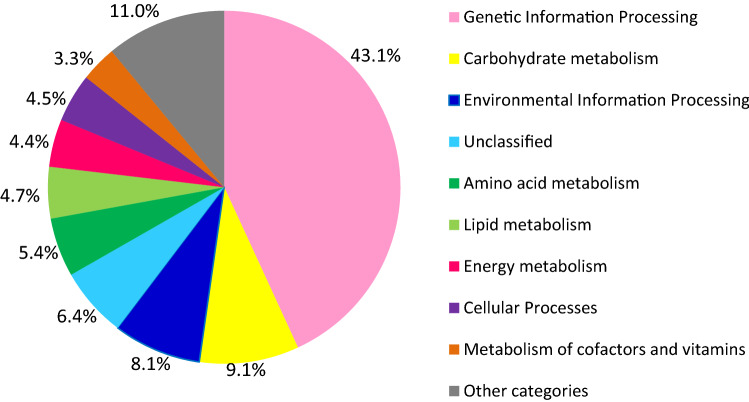
Annotation of *C. sorokiniana* transcripts based on functional categories KO database using KOALA tools at KEGG Web site^58^ (https://www.kegg.jp/blastkoala/).

In the subsequent functional analysis, the functional annotation was done based on the KO (KEGG Orthology) database using KOALA tools (available at KEGG Web site; https://www.kegg.jp) that assign the KO identifiers (K numbers) to the transcript by BLAST, and GHOSTX searchers. In this study, of the 18,310 transcripts that aligned with KO database, only 6971 transcripts (38.1%) were annotated, and had significant matches to different functional categories (Fig. 2). The highest number of genes identified was from the genetic information processing category, which were 3007 genes (43%). The second highest functional categories of transcripts identified were carbohydrate metabolism (637 genes), followed by environmental information processing (563 genes), amino acid metabolism (377 genes), lipid metabolism (329 genes), energy metabolism (304 genes), cellular processes (311 genes), and metabolism of cofactor, and vitamins (231 genes). The remaining 17% of KEGG annotated transcripts were from other categories.

**Figure 3:**
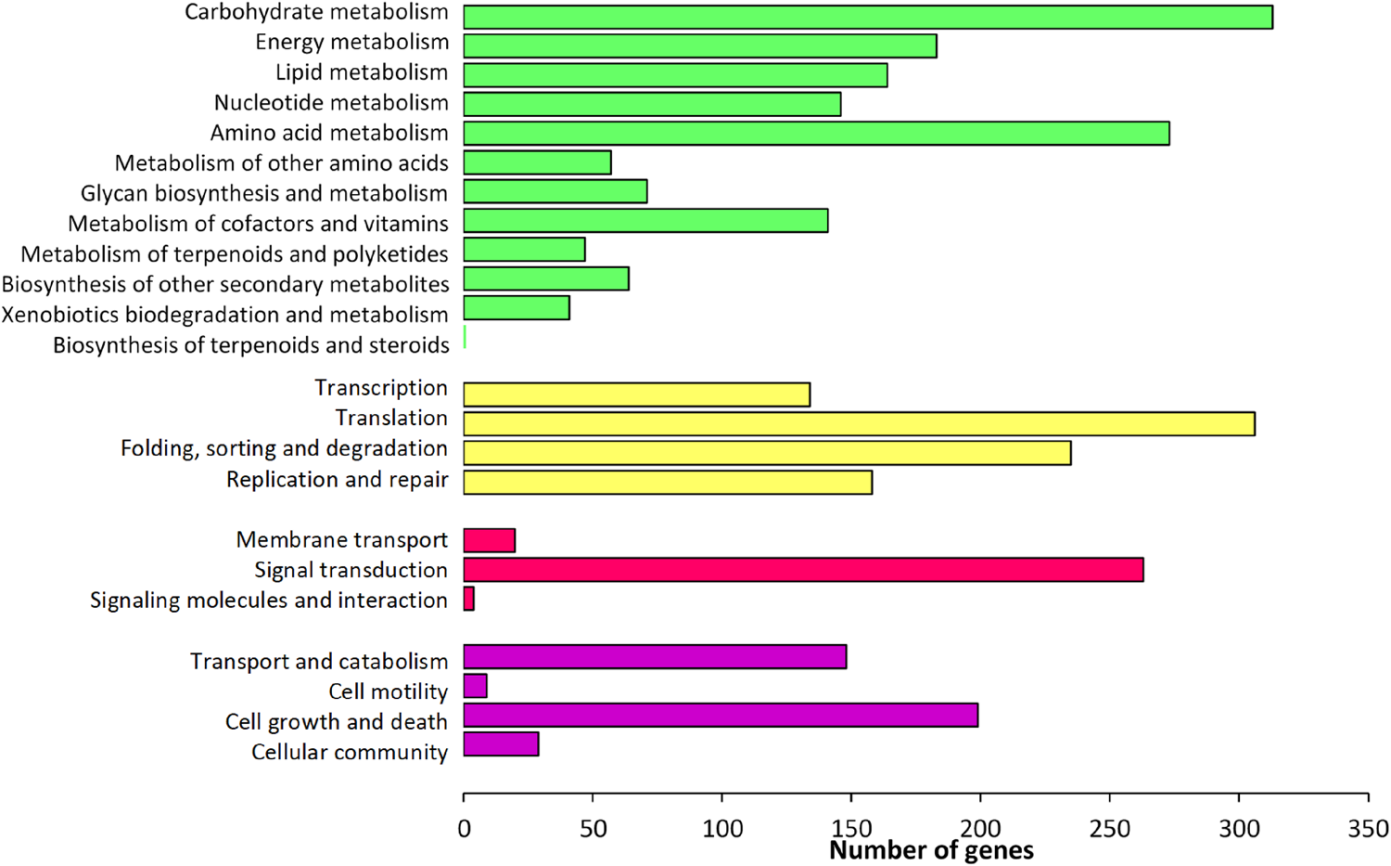
Functional classification, and pathway assignment of unigenes by KEGG Orthology (KO). The results are summarized in five main pathways modules: Metabolism (green); Genetic Information Processing (yellow); Environmental Information Processing (red); and Cellular Processes (purple). The y-axis indicates the name of the KEGG metabolic pathways. The x-axis indicates the number of genes annotated under the pathway.

Under pathway reconstruction of KO, each transcript from different functional categories was further elucidated into different metabolic pathways. All the metabolic pathways were then divided into four main pathway modules such as metabolism, genetic information processing, environmental information processing, and cellular processes (Fig. 3). According to this pathway assignment, 5018 transcripts (27.4% of all transcripts) were classified into 23 KEGG pathways. From the annotation result, the transcripts coding for all the genes related to the major metabolic pathways in *C. sorokiniana* were identified. The completeness of these reconstructed pathways indicates that the gene function assignments were biologically meaningful, and the EC number(s) has been correctly assigned to the annotated sequences (Table 4). Other KEGG pathways with multiple transcript hits encoding for a nearly complete pathway include nucleotide, and amino acid metabolism (such as serine, and threonine biosynthesis, lysine biosynthesis, and histidine biosynthesis), and cofactor, and vitamin biosynthesis (such as shikimate pathway, phenylalanine biosynthesis, coenzyme A biosynthesis, and biotin biosynthesis).

**Table 4:** Essential metabolic pathways annotated in the *C. sorokiniana* transcriptome.

Pathway	Number of genes found	Number of known genes^a^
Reductive pentose phosphate (Calvin-Bensen cycle)	11	12
Glycolysis/gluconeogenesis	10	10
Citrate cycle (TCA cycle)	9	10
Fatty acid biosynthesis	6	6
Triacylglycerol (TAG) biosynthesis	4	4
Starch biosynthesis	4	4
Isoprenoid biosynthesis (MEP pathway)	7	7

^a^Based on the KO annotation in KEGG database using BLASTKOALA, and published data in *C. sorokiniana,* and related green algae^21,44,65^.

### Differential expression analysis

**Figure 4:**
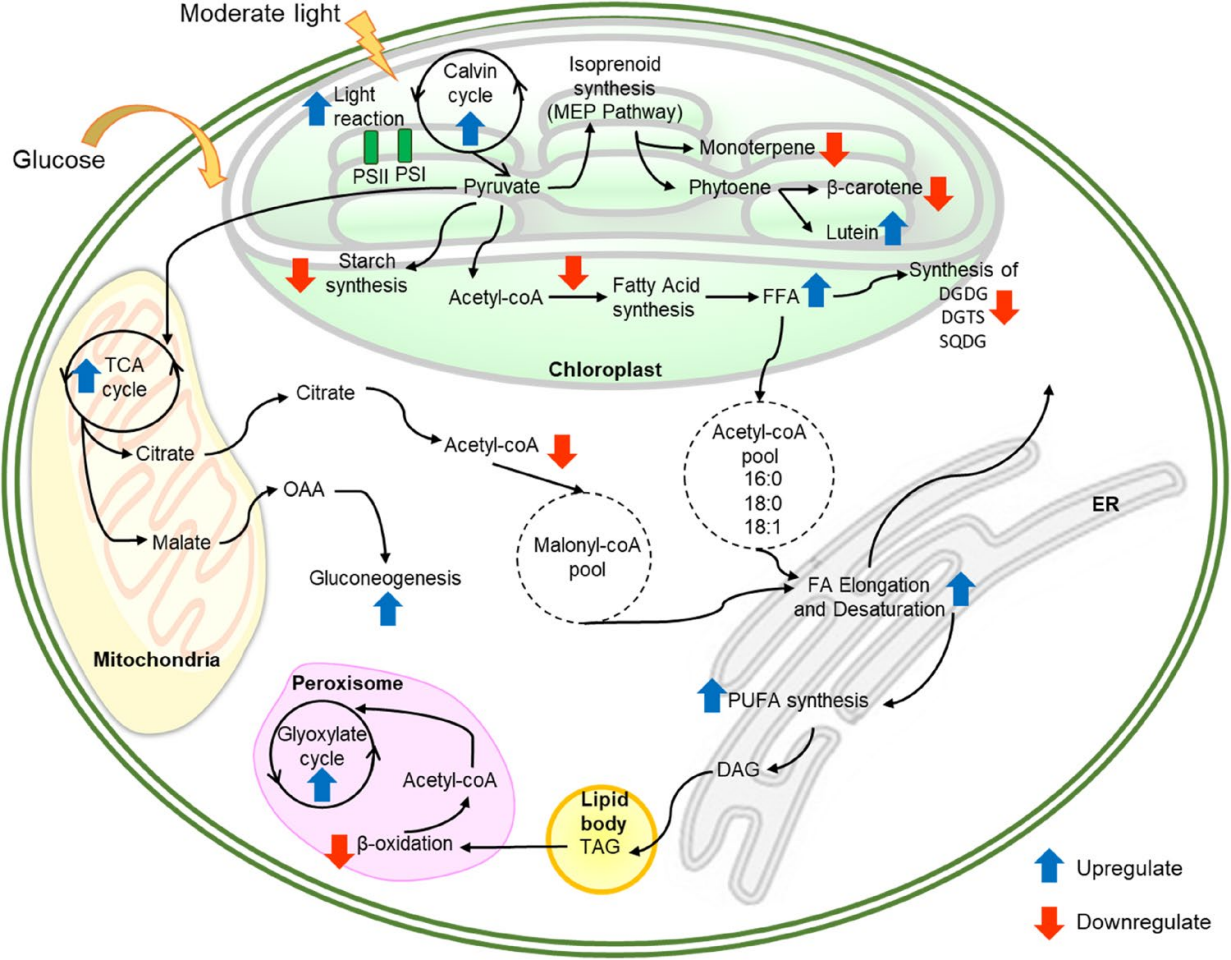
Overview of key metabolic pathways responsive to moderate light stress under nutrient-limiting conditions in *C. sorokiniana* Upregulation or downregulation of mRNA expression under stress condition based on transcriptomic analysis are indicated with blue upward arrows, and red downward arrows, respectively. *PSI* photosystem I, *PSII* photosystem II, *FFA* free fatty acid, *OAA* oxaloacetate, *FA* fatty acid, *PUFA* polyunsaturated fatty acid, *DGDG* digalactosyl diacylglycerol, *DGTS* diacylglyceryl *N*,*N*,*N*‐trimethyl homoserine, *SQDG* sulfoquinovosyl diacylglycerol, *TCA* tricarboxylic acid, *ER* endoplasmic reticulum.

Differential expression analysis between normal, and mixotrophic conditions revealed 2061 differentially expressed transcripts among the treatments, of which 937, and 1124 were upregulated, and downregulated in mixotrophic samples, respectively (Supplementary Data 1; Supplementary Figure S1, and S2). The biological pathways that were significantly affected (Adj. *P* < 0.05) by mixotrophic conditions are summarised in Fig. 4, and briefly described below. Detail information of genes involved in different metabolic pathways can be found in Supplementary Figures, and Tables.

### Secondary metabolism and carotenoids biosynthesis

Biosynthesis of secondary metabolites including carotenoids, phenols, and other isoprenoids originated from two pathways: mevalonate pathway, and non-mevalonate (or MEP) pathway. It is well known that carotenoids biosynthesis in *C. sorokiniana* is primarily biosynthesized via the plastid-localized non-mevalonate (or MEP/DOXP) pathway. All the key enzymes involved in the synthesis of important precursors for pigments biosynthesis, which are isopentenyl pyrophosphate (IPP), and dimethylallyl pyrophosphate (DMAPP) were identified (Supplementary Figure S3). The non-identified enzyme for mevalonate kinase (MVK, EC 2.7.1.36) confirmed that this pathway occurs in the plastids, not in the cytoplasm, which is similar to the other photosynthetic green algae^33^. Our transcriptome analysis revealed that the transcripts encoding genes potentially responsible for carotenoid production was generally upregulated when moderate light intensity, and glucose addition were applied to the *C. sorokiniana* culture (Table S1). This was evidenced by the upregulation of transcripts encoding 2-C-methyl-d-erythritol 4-phosphate cytidylyltransferase (CMS) or IspD; the gene that responsible for the production of an intermediate molecule from MEP to produce isopentenyl diphosphate (IPP), and dimethylallyl diphosphate (DMAPP). In the downstream step, lutein production was also upregulated (Fig. 4). This was evidenced by the upregulation of carotenoids lycopene epsilon cyclase, and ε-ring hydroxylase (Table S2). These two genes are required to cyclise the hydrocarbon chain, and hydroxylate the carotene rings to generate xanthophylls such as lutein, and zeaxanthin^34^. On the other hand, two genes related to the production of β-carotene, and astaxanthin were downregulated; they were phytoene dehydrogenase, and carotenoid cleavage dioxygenase^35^. Downregulation of these two genes showed that the mixotrophic condition was favourable forlutein production but not towards the biosynthesis of other carotenoids, and apocarotenoids (such as vitamin A, retinol, and abscisic acid hormone)^36^.

In the case of isoprenoid metabolism (see monoterpene biosynthesis Fig. 4), the downregulation of HMGS, the gene that converts cytosolic acetyl-CoA into HMG-CoA via the mevalonate pathways, is indicative of a general downregulation of the isoprenoid biosynthesis. Further downstream of this pathway showed downregulation of several genes such as those related with phenylpropanoid (lignin) biosynthesis which is cinnamyl alcohol dehydrogenase (CAD); gene related with tocopherol (vitamin E) biosynthesis which is MSBQ methyltransferase; and those related with monoterpenes biosynthesis which isogeranyl diphosphate (GPP) synthase (Supplementary Data 1, and Figure S4)^37^.

### Fatty acid biosynthesis, and lipid metabolism

The fatty acid biosynthesis in microalgae occurs in two subcellular components; first, de novo synthesis of fatty acids in plastids, and second, conversion into long-chain fatty acid occurs in the endoplasmic reticulum^38^. All transcripts encoding genes related to fatty acid biosynthesis, and TAG synthesis were detected in this *C. sorokiniana* transcriptome (Table 4). Under mixotrophic condition imposed in this study, the first step of fatty acid synthesis was downregulated, as detected by the downregulation of acetyl coA carboxylation reaction of both types of acetyl coA carboxylase (homomeric, and heteromeric) transcripts (Table S3). The plastidial acetyl coA carboxylase transcripts, whose products form a heteromeric multi-subunit enzyme complex that contains biotin carboxylase activity, whereas the cytosolic acetyl coA carboxylase is a homomeric multifunctional protein that does not contain biotin carboxylase activity^39^.

However, the subsequent steps in the lipid metabolism such as desaturation, and elongation processes were upregulated (Table S4–S6). This is evident by the upregulation of transcripts encoding acetyl coA ACP transacylase (FabH); ketoacyl ACP synthase (FabB, and FabF); oxoacyl ACP reductase (FabG); steryl-ACP desaturase (DESA1); stearoyl-coA desaturase (SCD or desC); and omega-6 desaturase (FAD2 or desA) in biosynthesis, and desaturation processes. DESA1, and desA responsible for desaturation step, and further exported to the cytosol into the acyl-coA, and acyl-lipid pools in the cytoplasm^38–40^. Meanwhile, elongation in endoplasmic reticulum showed upregulated as detected by ketoacyl coA synthase (KCS), acyl-coA reductase (FAR), very-long chain (3R)-hydroxyacyl-coA dehydratase (PAS1), and very long chain enoyl-coA reductase.

In the case of glycerolipid, and triacylglycerol (TAG) metabolism, most transcripts detected were downregulated, including, glycerol-3-phosphate *O*-acyltransferase (GPAT), lysophospholipid acyltransferase (LPLAT), TAG lipase (TagL), and diacylglycerol kinase (DGK). Several other transcripts encoding genes involved in the synthesis of membrane lipids especially thylakoid membrane such as UDP-sulfoquinovose synthase (SQD1), sulfolipid synthase (SQD2), phosphatidate cytidylyltransferase (CDS), cyclopropane-fatty-acyl-phospholipid synthase (CFAS), and phosphatidylcholinesterol *O*-acyltransferase (LCAT) were also downregulated (Table S7). All these transcripts involved in the formation of membrane components such as digalactosyl diacylglycerol (DGDG), diacylglyceryl *N*,*N*,*N*‐trimethyl homoserine (DGTS), and sulfoquinovosyl diacylglycerol (SQDG) (Fig. 4).

### Carbohydrate metabolism

Generally, most carbohydrate metabolic pathways (such as glycolysis, starch, and sucrose metabolisms) were downregulated (Table S9–S11). However, some transcripts involved in the glycolysis process were upregulated. For example, aldolase (ALDO), triose phosphate dehydrogenase (GAPDH), and phosphoglycerate kinase (PGK) showed upregulation. Whilst, the other transcript in all three pathways showed downregulation.

### Validation of gene expression through quantitative real-time PCR

**Figure 5:**
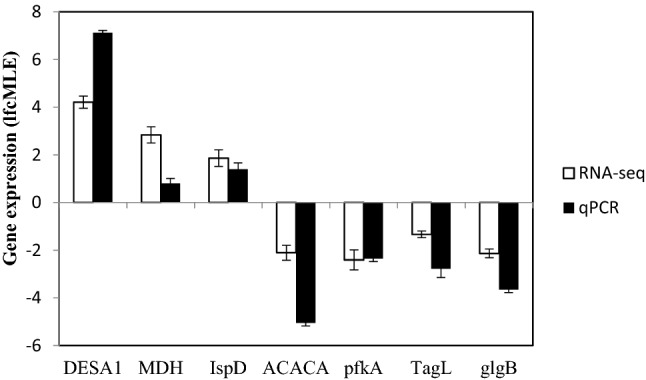
RT-qPCR validation of the gene expression patterns. The white bars represent the changes in the transcript from RNA-seq, and the black bars represent the relative expression level estimated by RT-qPCR. Whisker in each bar represents standard error.

Quantitative real-time PCR (RT-qPCR) was used to validate 7 differentially expressed genes identified by RNA-seq. The genes were chosen from each of the important pathways in the transcriptomic analysis of RNA-seq data. For example, stearyl-ACP desaturase (DESA1) from desaturation of fatty acid, malate dehydrogenase (MDH) from TCA cycle, 2-C-methyl-d-erythritol 4-phosphate cytidylyltransferase (IspD) from MEP pathway, Acetyl-Coa carboxylase (ACACA) from fatty acid biosynthesis pathway, TAG lipase (TagL) from TAG biosynthesis pathway, 1,4-glucan branching enzyme (glgB) from starch biosynthesis pathway, and phosphofruktokinase (pfkA) from glycolysis pathway. The primers used for each target genes, and selected housekeeping genes with their efficiency, and R^2^ data are shown in Table 5. In RNA-seq sequencing results, the expression levels of DESA1, MDH, and IspD were significantly upregulated, whilst the expression of ACACA, TagL, glgB, and pfkA were significantly downregulated in mixotrophic conditions. The gene expression profile based on RT-qPCR was highly correlated (R = 0.916) with RNA-seq confirming the reliability, and validity of the RNA-seq technique (Fig. 5).

**Table 5:** Housekeeping, and target genes, and their primers used for RT-qPCR.

Gene symbol	Target gene	Forward/reverse primers (5′–3′)	Product size (bp)	Efficiency value (%)	R^2^
RPL	Ribosomal protein L19	GTCTGGCTGGACCCCAATGA	143	90.4	0.999
		GCCTCTGCTGCGGTGC			
A-tub	Alpha-tubulin	CACAGTTTACCCGTCTCCCC	185	97.6	0.996
		TCAGCCGGTTAAGGTTGGTG			
PCNA	Proliferating cell nuclear antigen	TGGGCCTGAACCTGAACAAC	301	90.3	0.987
		TTGATGCCCTCCTTCGTCAC			
DESA 1	Stearyl-ACP desaturase	TGCTGGGTCGCTTCCTCTAC	397	94.3	0.995
		CCGTTGTTCAGCTCCGAGTG			
MDH	Malate dehydrogenase	TCACCAAGGAGGAGATTGAG	342	105.3	0.989
		CGCCTGTCTTAATGTTGGTC			
IspD	2-C-methyl-D-erythritol 4-phosphate cytidylyltransferase	CTGGTGGCAATTCACGACTC	263	99.3	0.981
		TCAGTGACCTCCAGCTTCTC			
ACACA	Acetyl Coa carboxylase	GCATGGCCGACCAGTTTGTG	347	94.1	0.966
		CCGGTCGTAGACATCAGGTG			
pfkA	Phosphofruktokinase	CAAGCCCATCACCCTTACCC	298	91.5	0.991
		CTCCACAGCCGTCTCAAACC			
TagL	TAG lipase	GCTGCTGGAACACCAGATGC	357	105.6	0.965
		GGCGCTGTCAGGTAGTTGAG			
glgB	1,4-glucan branching enzyme	GAGCGCGATGACTTTGGCAC	358	98.2	0.948
		TGGCGTTGTAGCCCAGCTTC

## Discussion

*Chlorella sorokiniana* can grow in various growth conditions making them a target organism for sustainable metabolite production. Findings from several microalgae have previously shown that mixotrophic conditions are more desirable to achieve both higher growth rates, and biomass yields (and even lipid content)^41,42^. In the present study, we induced the production of carotenoids, and lipids production through glucose assimilation, and light mixotrophic condition, and investigated the expression of genes involved in carotenoids, and lipid accumulation at the transcriptome level for the very first time. However, this transcriptomic analysis is not a substitute for detailed gene, and pathway studies, but it provides a broad overview of the important metabolic process from which to efficiently build a hypothesis that can guide future detailed studies on improving the carotenoid production, and lipid accumulation in this and others microalgae.

In the case of isoprenoids biosynthesis, there were two pathways responsible for the production of these biomolecules; the MVA, and MEP pathway. The incomplete genes for MVA pathway in this *C. sorokiniana* transcriptome suggest that the production of isoprenoids was dependent on MEP pathway that exists in the plastid. In our study, we found that homologs of the MVA pathway genes such as hydroxymethyl-glutaryl-CoA (HMG-coA) synthase (HMGS) exist in *C. sorokinina*. Conversely, homologs encoding hydroxymethyl-glutaryl-CoA reductase (HMGR), mevalonate kinase (MVK), phosphomevalonate kinase (PMK), and diphosphomevalonate decarboxylase (MVD) were absent. These results were proportional to the results of studies on *Ulva prolifera*^30^, *Porphyra umbilicalis*^29^, and *Chlorella zofingiensis*^40^. From the findings, the mixotrophic condition used in this study reduced the expression of sterols, sesquiterpenes, and triterpenes. These isoprenoids are secondary metabolites responsible to produce various bioactive compounds (such as antioxidant, antibacterial, antifungal, etc.) in microalgae^40^. The downregulation of the genes related to isoprenoids biosynthesis might explain our previous results regarding the level of phenolic compound, and their lowered antioxidant activity compared to normal growth condition^31^.

Meanwhile, for carotenoids biosynthesis located in the plastids showed that under mixotrophic condition lutein production was more favorable compared to the other carotenoids production. Our result was similar to the study done by Xiao et al*.*^18^, which they found two β-ring hydroxylases were upregulated in *Auxenochlorella protothecoides* culture that produced the highest lutein production. There are two routes leading to lutein production from α-carotene: β-ring hydroxylation to zeinoxanthin followed by ε-ring hydroxylation to lutein or ε-ring hydroxylation to α-cryptoxanthin, followed by β-ring hydroxylation to lutein^43^. In our transcriptome data, the second step after β-ring hydroxylation which is ε-ring hydroxylase was upregulated leading to lutein production was similarly found by Xiao et al*.*^18^. In terms of cultivation strategies, they used a two-step transition process from heterotrophic to photoautotrophic condition to increase lutein production—i.e. glucose as the carbon source in heterotrophic growth followed by illumination. The other study that used acetate as the carbon source in the mixotrophic growth of *C. sorokiniana*, however, their transcriptomic result obtained did not show any genes related to carotenoid production, either lutein or astaxanthin, being differentially expressed^44^. This indicates that glucose shows better results for lutein production compared to acetate. One study has also shown that glucose was the best carbon source for mixotrophic growth of all the *Chlorella* strains for increasing microalgal biomass, followed by glycerol, sodium acetate, and sucrose^12^. Furthermore, although genes related to the other carotenoid production such as β-carotene, and astaxanthin were detected in this *C. sorokinina* transcriptome, the mixotrophic condition was not suitable for their production. Thus, if pigments (such as β-carotene, and astaxanthin) are needed to be produced from this microalgae, different induction strategies will need to be ascertained.

In lipid metabolism, the downregulation of the first step in fatty acid synthesis followed by the upregulation of genes downstream of fatty acid synthesis, including elongation, and desaturation, showed contradictory results from one earlier study^21^. However, this pattern of regulation showed feedback inhibition of fatty acid synthesis like proposed by Andre et al*.*^45^. Our result was similar to Sirikhachornkit et al.^46^ who found the concomitant accumulation of TAG with downregulation of TAG, and DAG lipase genes under nitrogen deficiencies in *S. acutus*. Thus, these findings may provide a potential target for designing strategies for increasing fatty acid synthesis in microalgae. Meanwhile, the changes in the membrane lipid metabolism were due to the effect of carbon partitioning during mixotrophic conditions. This was discussed in detail by Alboresi et al*.*^47^, and Han et al*.*^48^ who investigated the effect of light, and nitrogen deprivation on *Nannochloropsis sp.* cultures using a combination of omics approach. In their studies, they found that chloroplast polar lipids were decreased, while the other membrane components were increased. This happened due to the accumulation of lipid accompanied by the regulation of inorganic phosphate transport across the chloroplast membranes, and tuning the carbon metabolic allocation between cell compartments such as cytoplasm, mitochondrion, and endoplasmic reticulum. Furthermore, the assimilation of glucose during mixotrophic conditions assisted the accumulation of lipids where the carbon skeleton from glycolysis, gluconeogenesis, and starch degradation might be directly channeled into fatty acids synthesis. The activities of enzymes involved in the starch synthesis pathway were down-regulated in this study supporting the hypothesis of starch to lipid shift under stress condition^49^. Since both synthesis of starch, and TAG share carbon precursors, blocking the starch synthesis can shift the carbon flux towards fatty acid, and TAG accumulation. Several other studies in different microalgae such as *C. reinhardtii*^50^, *Dunaliella tertiolecta*^51^, and *Scenedesmus obliquus*^52^, which have mutations that affected starch accumulation showed an increase in total lipid, and TAG contents under nitrogen deficiencies. In this study, lipids were stored in the form of polyunsaturated fatty acids which was similarly found by other researchers that also studied the effect of mixotrophic condition for biofuel production^53^. Many studies also reported that the microalgae would only produce either lipids or starch in order to increase the specific product yields. This was also seen in other microalgae such as *Chlamydomonas reinhardtii,* and *Miracritinum pusillum*^54–56^. On the other hand, repressing the β-oxidation is a clear strategy for maintaining a higher concentration of fatty acid within cells.

Transcriptomic analysis of *C. sorokiniana* under mixotrophic conditions enabled the exploration of a broad diversity of genes, and pathways. From the analysis, 18,310 assembled transcripts were obtained. The functional annotation, and classification of these transcripts provided a better understanding of *C. sorokiniana* transcriptome, and its molecular basis under mixotrophic conditions where glucose was added, and moderate light intensity were used. The findings point to several molecular mechanisms that potentially drive the overproduction of high-value metabolites such as carotenoids especially for lutein, unsaturated fatty acids synthesis, TAG accumulation. Meanwhile, the mixotrophic condition could repress the starch synthesis, sucrose synthesis, and isoprenoid biosynthesis. The transcriptome analysis supported the previous biochemical and morphological findings of *C. sorokiniana* cultured under photoautotrophic and mixotrophic conditions^31^.

## Materials and methods

### Strain

The *Chlorella sorokiniana* (NIES-2168) used in this study was obtained from the Marine Biotechnology Lab at the Faculty of Agriculture, Universiti Putra Malaysia, which had originally obtained from NIES (National Institute of Environmental Studies, Japan).

### Experimental design

The microalgae were cultured in Bold’s Basal Medium (BBM) (PhytoTechnology Laboratory®, USA). The stock medium was diluted from 50 × to 1 × using sterile distilled water, and adjusted to pH 6.6 using 1 M NaOH. Sterilisation was performed by autoclaving for 20 min at 121 °C. At the beginning of microalgae pre-culture preparation, the microalgae were first inoculated with 10% (vol/vol) of an exponentially growing culture, and allow to grow under continuous light with an intensity of approximately 10 µmol photons m^−1^ s^−1^ with a shaking speed of 30 rpm at 27 °C. These pre-culture microalgae were allowed to grow until mid-logarithmic phase at day 15, which produce approximately 2.5–3.0 × 10^6^ cells/mL. Then, the culture was divided into two flasks for normal, and mixotrophic conditions respectively. Each of the flasks contained 100 mL of 2 × 10^6^  cells/mL of *C. sorokiniana* culture. Cultures for the normal condition was allowed to grow under the same condition as pre-culture, whereas culture for mixotrophic condition was allowed to grow under moderate light intensity (100 µmol photons m^−1^ s^−1^), and supplemented with 2% glucose^31^. Both cultures were allowed to continue growing for 7 days. The experiments were conducted in a shaking incubator, and a conical flask was used as the growth chamber. A white fluorescence light source was located above the cultures. All experiments were repeated independently in triplicates. The microalgae were harvested by separating the pellet from the medium by centrifugation at 10,000 rpm for 10 min. The pellet was then flash-frozen using liquid nitrogen, and stored at − 20 °C prior to use.

### RNA extraction

Total RNA was extracted using TRIzol method. Briefly, 100 mg of frozen tissue was ground using a prechilled mortar, and pestle into a fine powder. 1 mL TRIzol reagent (Invitrogen, California) was added to the ground tissue, and homogenisation continued until no visible debri remains. The homogenised samples were incubated at room temperature for 5 min. Chloroform with 0.2 times the volume of Trizol solution was added to the solution, and the mixture was vortexed vigorously for about 15 s, and incubated at room temperature for 2 to 3 min. After centrifugation, the aqueous layer was transferred to a column of RNeasy from a mini RNA isolation kit (Qiagen, Germany) for further purification. The residual DNA was eliminated by performing a column DNase digestion at 37 °C for 30 min. The integrity of the extracted RNA was determined by gel electrophoresis, and its concentration was measured using a biospectrometer (Eppendorf, Germany).

### Library preparation, and Illumina sequencing

The library for RNA-sequencing was prepared using NEBNext Ultra Directional RNA Library Prep kit for Illumina from New England Biolabs (NEB, UK) according to the manufacturer's protocol. The molarity, and libraries sizes were assessed on an Agilent 2100 Bioanalyzer (Agilent Technologies, Germany). Sequencing of 100 bp paired-end reads was performed on the Illumina HiSeq 2000 at HIR Central Lab High End Instrument, University of Malaya, Malaysia.

### De novo* transcriptome assembly, and annotation*

Filtered paired-end reads of each sample were pooled, separated into their respective orientation, and collapsed further to retain only the unique non-redundant reads using the FASTX-toolkit (https://hannonlab.cshl.edu/fastx_toolkit/). The transcriptome de novo assembly was performed using Trinity (version 2.0.6) with default settings^32^. Gene predictions, and protein-coding potential of the de novo assembled transcripts were determined using AUGUSTUS (version 2.5.5)^57^. To obtain an accurate gene model, the *Chlamydomonas reinhardtii* sequence (genes, and proteins), and gene structure was used to train AUGUSTUS. A comprehensive annotation of the protein-coding gene, and protein repertoire were performed using BLASTN, and BLASTX, respectively against the NCBI non-redundant, SwissProt (and UniProt), Refseq, KEGG (Kyoto Encyclopedia of Genes, and Genomes)^58^, and GO (Gene Ontology) databases using a threshold of E-value < 10^–5^ to define significant hits, and for ascribing potential gene function. Functional MapMan BIN categories were ascribed to predicted genes using the Mercator^59^. Default parameters were used with additional ‘CHLAMY’, and ‘IPR’ settings enabled.

### Differential gene expression, and functional enrichment analysis

The alignment of filtered paired-end reads against the predicted protein-coding genes was performed using Subread aligner^60^, and counting of reads (read summarization) was performed with Feature Counts^61^ with default settings in R software^62^. Differential gene expression analysis was carried out using DESeq2^63^, and genes were defined as significantly different between treatment conditions, and controls at a false discovery rate (FDR) < 0.05. Transcript abundance was estimated using DESeq2 using the variance stabilization normalization (VST). Enrichment of MapMan BINs categories was determined by Fisher’s exact test adjusted with Bonferonni correction for multiple testing correction using the Corto tool^64^. MapMan BIN categories were deemed significantly enriched at an adjusted *P* value < 0.05.

### Quantitative real-time PCR (RT-qPCR) validation

Quantitative real-time PCR (RT-qPCR) was carried out to validate the results of the RNA-seq. Total RNA was extracted using TRIzol method as described for RNA extraction. The reverse transcription step for the preparation of the cDNA library was performed using SensiFAST cDNA Synthesis Kit (Bioline, UK) according to the manufacturer’s instructions. Three-hundred nanograms of the RNA template from each sample were converted into cDNA in 20 µL. The control was prepared by using a similar amount of total RNA that was subjected to the cDNA synthesis reaction without the presence of reverse transcriptase. A 300 ng cDNA template pool produced from the reverse transcription reaction was subsequently used to assess housekeeping genes, and target genes transcript levels in real-time PCR assays. The information of primers used for housekeeping genes, and target genes are presented in Table 5.

## Supplementary information

Supplementary Information 1.

Supplementary Information 2.

## Data Availability

The authors declare that all the data in this manuscript are available.
