# Supplementary material for: De novo transcriptome analysis of Chlorella sorokiniana: effect of glucose assimilation, and moderate light intensity
Source: Sci Rep. 2020 Oct 15;10:17331. doi: 10.1038/s41598-020-74410-4 (PMC7562877; doi:10.1038/s41598-020-74410-4)
Supplement: Supplementary file 1 — Supplementary Information 1. [file 41598_2020_74410_MOESM1_ESM.docx]

**De Novo transcriptome analysis of Chlorella sorokiniana: Effect of glucose assimilation and moderate light intensity**

Siti Nor Ani Azaman^1^, Darren C. J. Wong^2^, Sheau Wei Tan^3^, Fatimah M. Yusoff^4,5^, Norio Nagao^4,6^, Swee Keong Yeap^7^

^1^Centre of Foundation Studies for Agricultural Sciences, Universiti Putra Malaysia, Serdang, Selangor, Malaysia

^2^Ecology and Evolution, Research School of Biology, The Australian National University, Acton, Australia

^3^Laboratory of Immunotherapeutics and Vaccines, Institute of Bioscience, Universiti Putra Malaysia, Serdang, Selangor, Malaysia

^4^Laboratory of Marine Biotechnology, Institute of Bioscience, Universiti Putra Malaysia, Serdang, Selangor, Malaysia

^5^Department of Aquaculture, Faculty of Agriculture, Universiti Putra Malaysia

^6^102 Naname-go, Shinkamigoto-cho, Minami Matsuura-gun, Nagasaki 857-4214, Japan

^7^China-ASEAN College of Marine Sciences, Xiamen University Malaysia, Sepang, Selangor, Malaysia

*Correspondence should be addressed to Swee Keong Yeap: skyeap2005@gmail.com

**Figure S1**

Figure S1. MA-plot contrasting gene expression levels between normal and stress conditions based on normalized counts. The plot depicts the shrinkage of log_2_ fold changes resulting from the incorporation of zero-centered normal prior. Red and grey points are DE and non-DE transcripts, respectively. Triangles depict shrunken log_2_ fold change point a greater and/or lesser than -3 and 3 log_2_ fold change. Each dot in the MA plot corresponds to a gene. The x-axis represented the mean expression which corresponds to the average counts adjusted by library size factors (normalization constant) of the sample, and y-axis represented the log_2_ fold change, which describes how much a quantity changes from one condition to another.

**Figure S2**

Figure S2. Functional categories of differentially expressed genes based on annotation with KEGG database. The blue bars represent the upregulated DEGs, the red bars represent the downregulated DEGs in stress conditions.


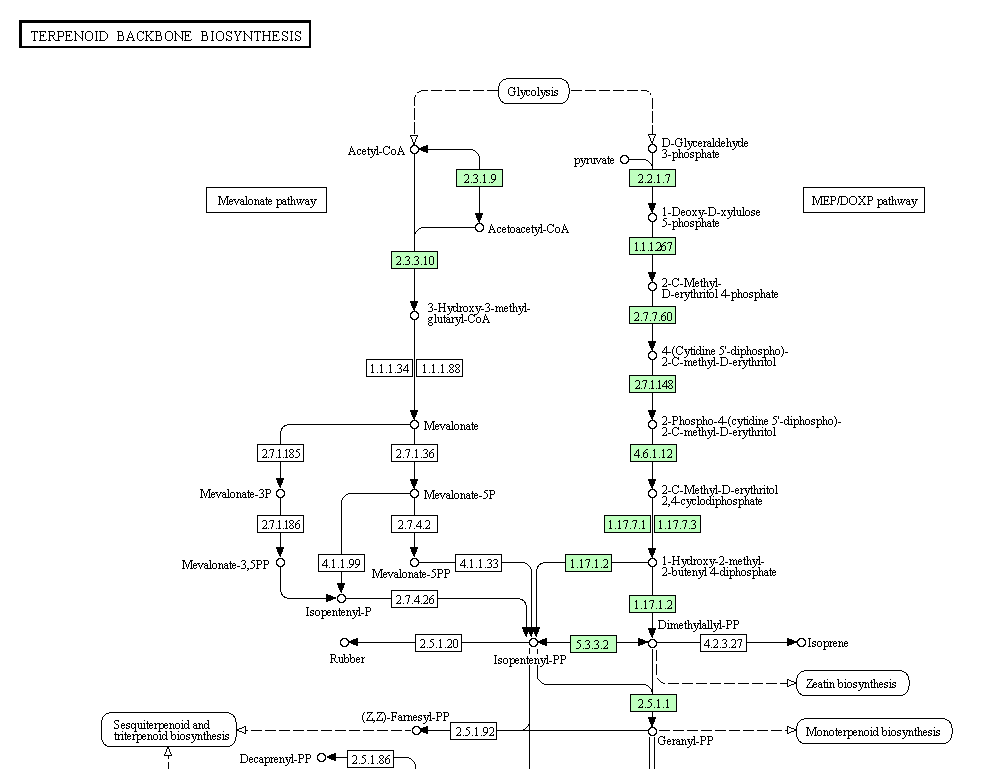

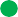


## Figure S3. Biosynthetic pathways for the generation of the isoprenoid building units, isopentenyl diphosphate (IPP) and dimethylallyl diphosphate (DMAPP) (highlighted in red box) reconstructed based on the *de novo* assembly and annotation of *C. sorokiniana* transcriptome. Green dot represents biomolecules, while green box highlighted the enzymes found in the *C. sorokiniana* transcriptome.

Table S1. Non Mevalonate (or MEP) pathway

| **Enzyme** | **EC number** | **Gene name** | **Transcript found** | **Log^2^ fold change** | **Modulation** |
| --- | --- | --- | --- | --- | --- |
| DOXP synthase | EC 2.2.1.7 | Dxs | 5 (g10694, g10696, g10698, g10700, g10702) | X | X |
| DOXP reductase | EC 1.1.1.267 | IspC | 2 (g16125, g16126) | X | X |
| 2-C-methyl-D-erythritol 4-phosphate cytidylyltransferase | EC 2.7.7.60 | IspD | 1(g17151) | 1.86231 | UP |
| 4-diphosphocytidyl-2-C-methyl-D-erythritol kinase | EC 2.7.1.148 | IspE | 1(g14585) | X | X |
| 2-C-methyl-D-erythritol 2,4-cyclodiphosphate synthase | EC 4.6.1.12 | IspF | 2 (g11740, g11741) | X | X |
| HMB-PP synthase | EC 1.17.7.1 ,  EC 1.17.7.3 | IspG | 1(g9697) | 0.408189 | UP |
| HMB-PP reductase | EC 1.17.1.2 | IspH | 1(g8508) | X | X |
| isopentenyl-diphosphate delta-isomerase | EC:5.3.3.2 | idi, IDI | 1(g3291) | X | X |

Table S2. Carotenoid biosynthesis and catabolism pathway

| **Enzyme** | **EC number** | **Gene name** | **Transcript found** | **Log^2^ fold change** | **Modulation** |
| --- | --- | --- | --- | --- | --- |
| Zeta-carotene desaturase | EC:1.3.5.6 | ZDS, crtQ | 2 (g16142, g16143) | X | X |
| Phytoene synthase | EC:2.5.1.32  EC 2.5.1.99 | crtB | 1 (g9836) | X | X |
| 15-cis-phytoene desaturase | EC:1.3.5.5 | PDS, crtP | 2 (g17941, g17944) | X | X |
| Lycopene beta-cyclase | EC:5.5.1.19 | lcyB, crtL1, crtY | 2 (g6623, g6625) | X | X |
| Lycopene epsilon-cyclase | EC:5.5.1.18 | lcyE, crtL2 | 1 (g10098) | 1.043778 | UP |
| Prolycopene isomerase | EC:5.2.1.13 | crtISO, crtH | 1 (g9191) | X | X |
| Zeaxanthin epoxidase | EC:1.14.15.21 | ZEP, ABA1 | 1 (g12090) | X | X |
| 9-cis-epoxycarotenoid dioxygenase | EC:1.13.11.51 | NCED | 1 (g11033) | X | X |
| Lycopene cyclase CruA | EC:5.5.1.19 | cruA | 1 (g3702) | X | X |
| Zeta-carotene isomerase | EC:5.2.1.12 | Z-ISO | 1 (g5452) | X | X |
| Beta-carotene 3-hydroxylase | EC:1.14.13.129 | crtZ | 1 (g7095) | X | X |
| Beta-ring hydroxylase | EC:1.14.-.- | LUT5, CYP97A3 | 1 (g5043) | X | X |
| Epsilon ring hydroxylase | EC 1.14.99.45 | LUT1, CYP97C1 | 1 (g7094) | 0.979892 | UP |
| Carlactone synthase | EC:1.13.11.69  EC 1.13.11.70 | CCD8 | 1 (g9055) | X | X |
| Phytoene dehydrogenase | EC:1.3.99.- | crtD | 1 (g10417) | -0.89393 | DOWN |
| Carotenoid cleavage dioxygenase | EC:1.14.99.n4 | CCD1 | 1 (g8971) | -1.45354 | DOWN |
|  |  |  |  |  |  |


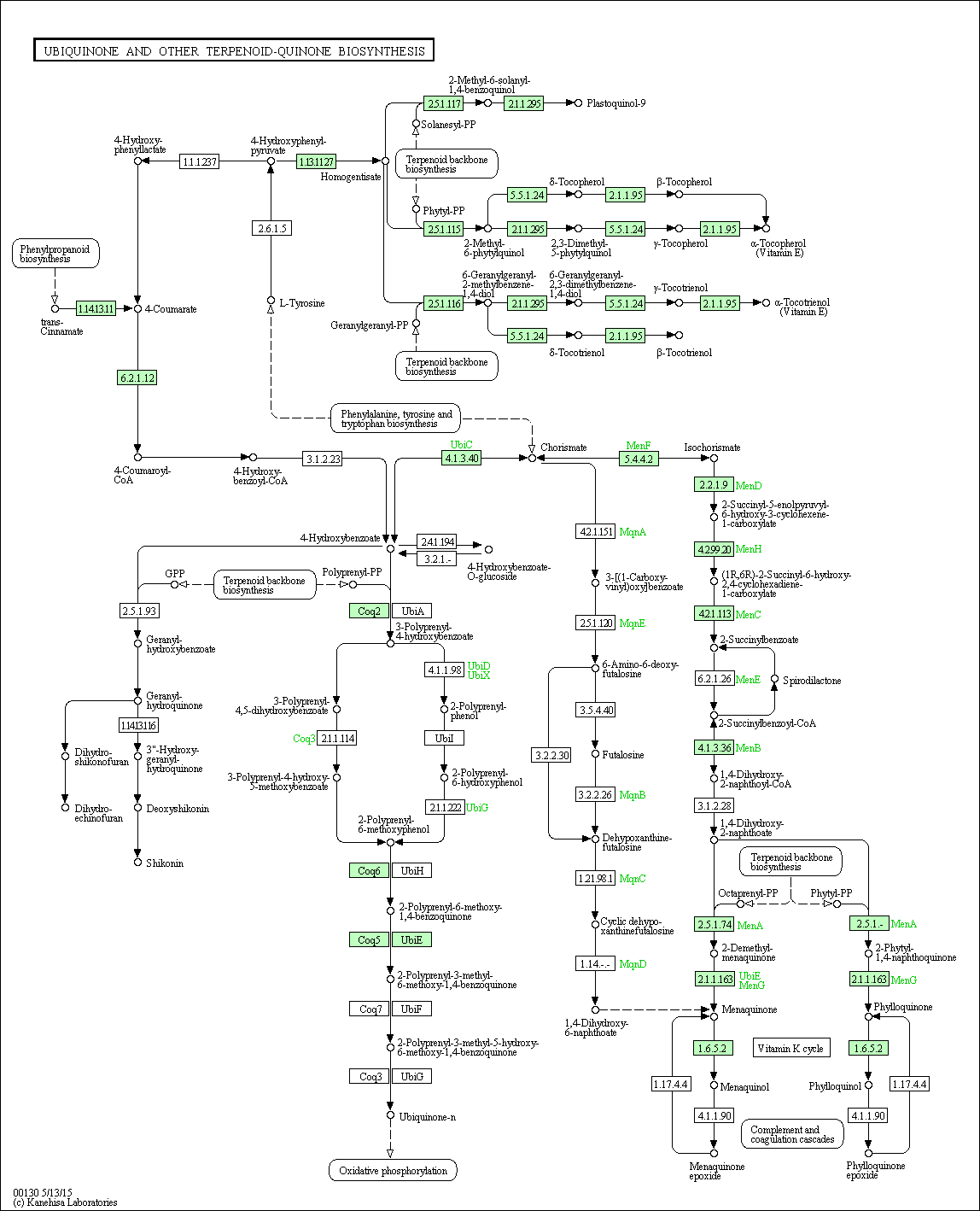


Figure S4. Biosynthetic pathways for the generation of the isoprenoid and other terpenoids, such as phenylpropanoids (lignin) and tocopherols (vitamin E). Green box highlighted the enzymes found in the *C. sorokiniana* transcriptome.

Table S3. Fatty acid biosynthesis pathway

| **Enzyme** | **EC number** | **Gene name** | **Transcript found** | **Log^2^ fold change** | **Modulation** |
| --- | --- | --- | --- | --- | --- |
| Acetyl Coa carboxylase (plastid and cytosol) | EC 6.4.1.2 | ACACA | 8 (g6564, g9695, g16344, g9538, g5870, g7112, g8672, g8673 | -2.10657 | DOWN |
| Acetyl CoA ACP transacylase | EC 2.3.1.38,  EC 2.3.1.39 | ACAT, KASIII, FabH | 1 (g6303) | 1.13192 | UP |
| Malonyl CoA:ACP transacylase | EC 2.3.1.39 | FAS, FabD | 2 (g3184, g3185) | X | X |
| 3-ketoacyl-ACP synthase | EC 2.3.1.41 @ EC 2.3.1.179 | KASI, KASII, FabB, FabF | 4 (g1456, g1459, g13471, g14611) | 1.728205 | UP |
| Oxoacyl-ACP reductase | EC 1.1.1.100 | FAS2, FabG | 3 (g15111) | 5.024732 | UP |
| 3-Hydroxyacyl ACP dehydrase | EC 4.2.1.17 | ECH, FabZ | 1 (g10965) | X | X |
| Enoyl-ACP reductase | EC 1.3.1.9 | EAR, FabI | 2 (g16371, g16373) | X | X |
| Fatty acyl-ACP thioesterase A | EC 3.1.2.14 | FATA, FatB | 1 (g6994) | X | X |


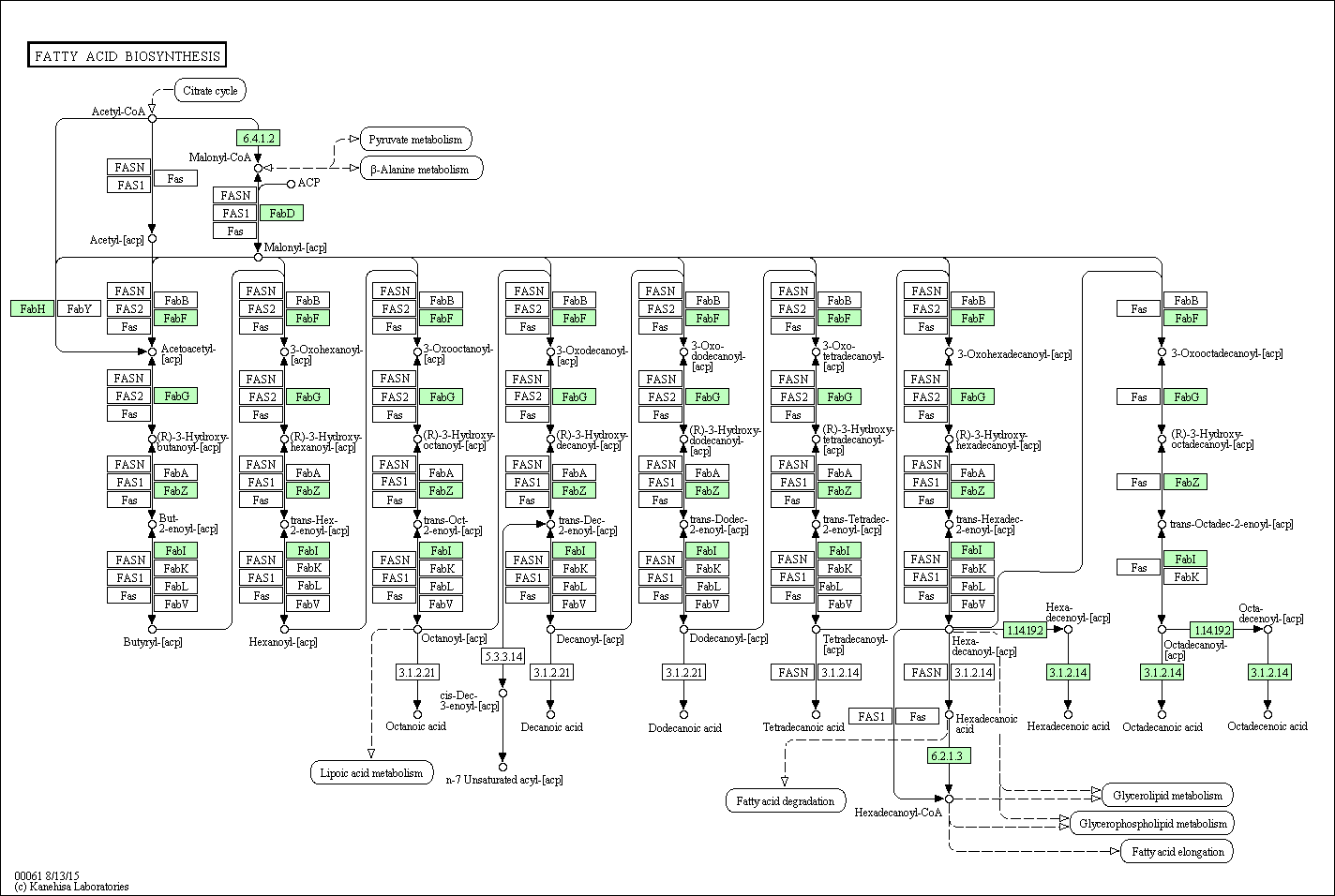


Figure S5. Fatty acid biosynthetic pathways in *C. sorokininana*. Green box highlighted the enzymes found in the *C. sorokiniana* transcriptome.

Table S4. Desaturation of fatty acid

| **Enzyme** | **EC number** | **Gene name** | **Transcript found** | **Log^2^ fold change** | **Modulation** |
| --- | --- | --- | --- | --- | --- |
| Stearyl-ACP desaturase, Δ9-D | EC 1.14.19.2 | DESA1 | 6 (g9907, g9908, g9910, g9912, g9914, g9916) | 4.211031 | UP |
| Stearoyl-CoA desaturase (delta-9 desaturase) | EC:1.14.19.1 | SCD, desC | 2 (g867, g6951) | 0.83664 | UP |
| Omega-6 desaturase (delta-12 desaturase) | EC:1.14.19.- | FAD2, desA | 1 (g6334) | 1.1298 | UP |
| Omega-3 fatty acid desaturase (delta-15 desaturase) | EC:1.14.19.- | FAD8, desB | 1 (g4534) | x | x |

Table S5. Elongation in mitochondria (2 block missing)

| **Enzyme** | **EC number** | **Gene name** | **Transcript found** | **Log^2^ fold change** | **Modulation** |
| --- | --- | --- | --- | --- | --- |
| Mitochondrial trans-2-enoyl-CoA reductase | EC 1.3.1.38 | MECR, NRBF1 | 6 (g3628, g3629, g12758, g12759, g12760, g12761) | -1.44502 | DOWN |

Table S6. Elongation in Endoplasmic reticulum (ER)

| **Enzyme** | **EC number** | **Gene name** | **Transcript found** | **Log^2^ fold change** | **Modulation** |
| --- | --- | --- | --- | --- | --- |
| very-long-chain β-ketoacyl-CoA synthase | EC:2.3.1.199 | KCS | 8 (g1019, g1021, g1917, g1918, g1919, g5537, g13030, g15566) | 0.499532 | UP |
| 17beta-estradiol 17-dehydrogenase / very-long-chain 3-oxoacyl-CoA reductase | EC:1.1.1.62 | KAR, IFA38 | 1 (g17572) | X | X |
| Acyl-coA reductase | EC 1.2.1.50 | FAR | 1 (g10097) | 1.8856 | UP |
| a very-long-chain acyl-CoA | EC:4.2.1.134 | PHS1, PAS2 | 4 (g4472, g4473, g16144, g16145) | 1.695083 | UP |
| Very-long-chain enoyl-CoA reductase | EC:1.3.1.93 | TER, TSC13, CER10 | 3 (g3299, g3301, g3303) | 1.611245 | UP |
| 3-hydroxyacyl-CoA dehydrogenase / enoyl-CoA hydratase / 3-hydroxybutyryl-CoA epimerase | EC:1.1.1.35,  EC 4.2.1.17,  EC 5.1.2.3 | fadJ | 1 (g12318) | X | X |

Table S7. Glycerolipid and triacylglycerol (TAG) metabolism

| **Enzyme** | **EC number** | **gene name** | **Transcript found** | **Log^2^ fold change** | **Modulation** |
| --- | --- | --- | --- | --- | --- |
| Glycerol kinase | EC 2.7.1.30 | GK | 2 (g14171, g14172) | X | X |
| Glycerol-3-phosphate O-acyltransferase | EC:2.3.1.15 | GPAT | 2 (g3177, g3178) | -1.5154 | DOWN |
| 1-Acyl-sn-glycerol-3-phosphate O-acyltransferase | EC:2.3.1.51 | AGPAT | 1 (g876) | X | X |
| Phosphatidate phosphatase | EC:3.1.3.4 | PAP | 2 (g11288, g11292) | X | X |
| Diacylglycerol O-acyltransferase | EC:2.3.1.20 | DGAT | 3 (g8796, g9610, g9612) | 1.00958 | UP |
| Lysophospholipid acyltransferase | EC 2.3.1.22 | LPLAT | 1 (g3747, g3749) | -1.08845 | DOWN |
| TAG lipase | EC:3.1.1.3 | TagL | 12 (g7347, g7348, g7349, g13552, g13553, g13554, g13555, g13556, g13557, g13558, g13559, g13560) | -1.3344 | DOWN |
| Alpha-D-galactosidase | EC 3.2.1.22 |  | 3 (g13175, g13176, g13177) | X | X |
| Diacylglycerol kinase | EC 2.7.1.107 | DGK | 8 (g12738, g12739, g1540, g1541, g624, g626, g627, g9618) | -0.8426 | DOWN |
| UDP-sulfoquinovose synthase | EC 3.13.1.1 | SQD1 | 2 (g10548 and g10549) | -1.52188 | DOWN |
| Sulfolipid synthase | EC 3.13.1.1 | SQD2 | 2 (g4276, g4277) | -1.06168 | DOWN |
| Phosphatidate cytidylyltransferase | EC 2.7. 7.41 | CDS | 2 (g1645 and g1646) | -2.02719 | DOWN |
| Cyclopropane-fatty-acyl-phospholipid synthase | EC 2.1.1.79 | CFAS | 1 (g7076) | -1.43698 | DOWN |
| Phosphatidylcholinesterol O-acyltransferase | EC 2.3.1.43 | LCAT | 1 (g8168) | -1.45559 | DOWN |


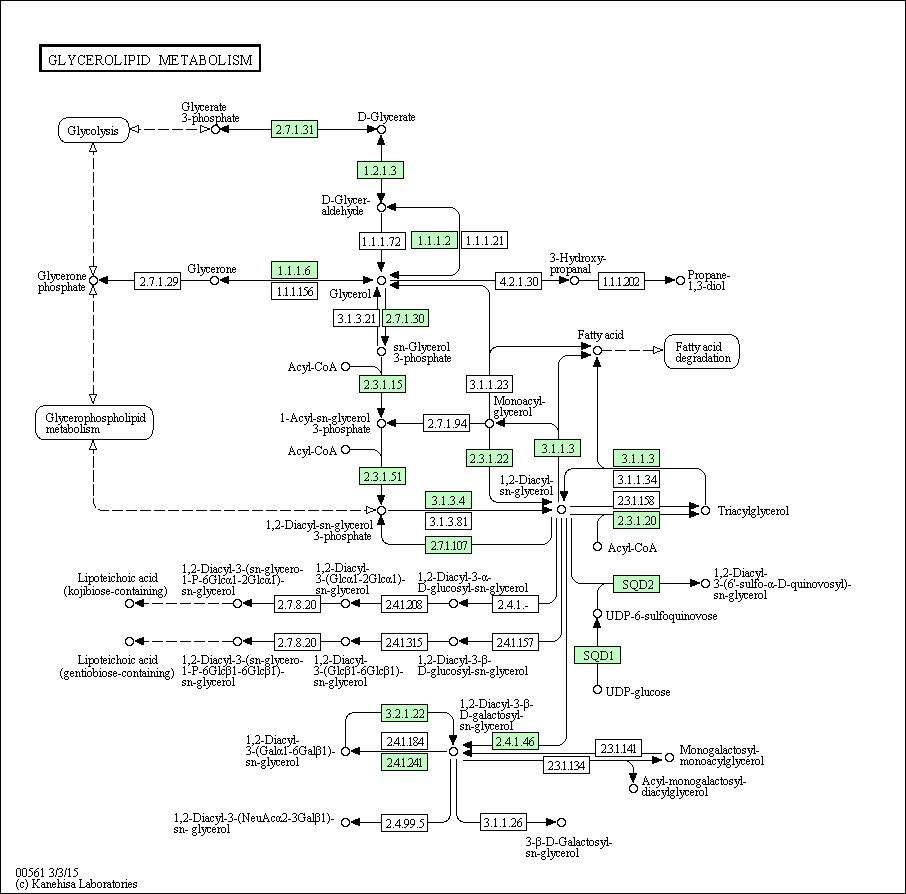


Figure S6. Glycerolipid metabolism pathway. Green box highlighted the enzymes found in the *C. sorokiniana* transcriptome.

Table S9. Glycolysis

| **Enzyme** | **EC number** | **Gene name** | **Transcript found** | **Log^2^ fold change** | **Modulation** |
| --- | --- | --- | --- | --- | --- |
| Hexokinase | EC 2.7.1.1 | Hex | 3 (g959, g960,g961) | X | X |
| Phosphoglucoisomerase | EC 5.3.1.9 | GPI | 4 (g3646, g3648, g6310, g7399) | X | X |
| Phosphofructokinase | EC 2.7.1.11 | pfkA | 15 (g10374, g10376, g10398, g11261, g11263, g11265, g13382, g13383, g13384, g13385, g13939, g13940, g13941, g13942, g16399), | -2.40784 | DOWN |
| Aldolase | EC 4.1.2.13 | ALDO | 8 (g1130, g4875, g4876, g4877, g4981, g4982, g4984, g11247) | 1.097251 | UP |
| Isomerase | EC 5.3.1.1 | TPI | 2 (g4925, g11007) | X | X |
| Triose phosphate dehydrogenase | EC 1.2.1.12 | GAPDH | 4 (g1136, g5927, g5928, g17106) | 1.29015 | UP |
| Phosphoglycerate kinase | EC 2.7.2.3 | PGK | 6 (g11168, g11169, g11170, g11171, g11172, g11173) | 0.9602 | UP |
| Phosphoglycerate mutase | EC 5.4.2.12 | iPGM | 9 (g15159, g15160, g15161, g15162, g15163, g6647, g6648, g16256, g16258), | -2.39275 | DOWN |
| Enolase | EC 4.2.1.11 | ENO | 1(g16192) | X | X |
| Pyruvate Kinase | EC 2.7.1.40 | PK | 11(g68, g69, g5103, g6705, g10248, g10249, g12717, g14677, g14678, g17852, g17853) | -2.29778 | DOWN |

Table S10. Starch metabolism

| **Enzyme** | **EC number** | **Gene name** | **Transcript found** | **Log^2^ fold change** | **Modulation** |
| --- | --- | --- | --- | --- | --- |
| Phosphoglucomutase | EC 5.4.2.2 | pgm | 1 (g17215) | X | X |
| Glucose-1-phosphate adenylyltransferase or ADP glucose pyrophosphorylase | EC 2.7.7.27 | glgC | 5 (g3904, g5908, g5909, g7113, g7116) | X | X |
| Starch synthase | EC 2.4.1.21 | glgA | 9 (g248, g249, g3372, g3376, g3380, g3384, g3388, g3390, g4463) | -1.15037 | DOWN |
| 1,4-alpha-glucan branching enzyme | EC 2.4.1.18 | glgB | 8 (g6526, g10852, g10853, g10854, g10855, g10856, g10857, g10977) | -2.13615 | DOWN |
| Alpha-amylase | EC 3.2.1.1 | amyA, malS | 2 (g10293, g10295) | -1.514258681 | DOWN |
| Beta-amylase | EC 3.2.1.2 |  | 5 (g1864, g1865, g6070, g6071, g12914) | -0.668340393 | DOWN |
| Starch phosphorylase | EC 2.4.1.1 | glgP, PYG | 2 (g4382, g4384) | X | X |
| 4-alpha-glucanotransferase/ debranching enzyme maltodextrin glycosyltransferase | EC 2.4.1.25 | malQ | 14 (g1196, g1199, g1204, g1211, g1216, g1219, g1222, g1225, g1232, g1235, g1238, g1241, g18264, g18267) | X | X |

Table S11. Sucrose metabolism

| **Enzyme** | **EC number** | **Gene name** | **Transcript found** | **Log^2^ fold change** | **Modulation** |
| --- | --- | --- | --- | --- | --- |
| Sucrose synthase |  | SuSy | 2 (g11797, g11799) | -2.08329 | DOWN |
| Sucrose phosphate synthase |  | SPS | 4 (g3217, g3995, g5522, g5523) | -1.12226 | DOWN |
| Sucrose phosphate phosphatase |  | SPP | 2 (g6877, g8208) | -1.144195799 | DOWN |
| UDP-glucose pyrophosphorylase |  | UDPGP | 2 (g16345, g4502) | X | X |
| Fructokinase |  |  | 2 (g11029, g13328) | X | X |
| Hexokinase |  |  | 3 (g959, g960, g961) | X | X |
| Invertase |  |  | 4 (g15622, g2760, g2761, g7508) | -1.740124813 | DOWN |
|  |  |  |  |  |  |
